# Impact of autologous platelet rich plasma use on postoperative acute kidney injury in type A acute aortic dissection repair: a retrospective cohort analysis

**DOI:** 10.1186/s13019-020-01383-w

**Published:** 2021-01-07

**Authors:** Jiaqi Tong, Liang Cao, Liwei Liu, Mu Jin

**Affiliations:** 1 Department of Anesthesiology, Beijing Friendship Hospital, Capital Medical University, No. 95 Yongan Rd, Xicheng District, Beijing City, 100050 China; 2 Department of Anesthesiology, Beijing Anzhen Hospital, Capital Medical University, Beijing Institute of Heart Lung and Blood Vessel Diseases, and Beijing Engineering Research Center of Vascular Prostheses, No. 2 Anzhen Rd, Beijing, 100029 China; 3 Department of Anesthesiology, Fuwai Hospital, National Center for Cardiovascular Diseases, Peking Union Medical College and Chinese Academy of Medical Sciences, No.167, Beilishi Road, Xicheng District, Beijing, 100037 China

**Keywords:** Autologous platelet rich plasma, Acute aortic dissection, Acute kidney injury

## Abstract

**Background:**

Perioperative coagulopathy and blood transfusion are common in patients undergoing Stanford type A acute aortic dissection (AAD) repair. The autologous platelet-rich plasmapheresis (aPRP) technique is a blood conservation approach to reduce blood transfusions and morbidity in patients at high risk of bleeding. The purpose of this study was to analyze the effect of aPRP on outcomes, especially in postoperative acute kidney injury (post-AKI), in patients undergoing AAD surgery.

**Methods:**

Six hundred sixty patients were divided into aPRP and non-aPRP groups according to aPRP use. The primary endpoint was the difference in the incidence of post-AKI between two groups. The secondary endpoints were risk factors for post-AKI and to assess clinical outcomes. The risk factors associated with post-AKI were calculated, and all outcomes were adjusted by propensity-score matching analysis.

**Results:**

A total of 272 patients (41.2%) received aPRP, whereas 388 were in the non-aPRP group. Compared to non-aPRP group, the occurrence of post-AKI increased by 14.1% (*p* = 0.002) and 11.1% (*p* = 0.010) with and without propensity adjustment in the aPRP group, respectively. The aPRP group required fewer intraoperative transfusions (*p* < 0.05) and shortened the duration of mechanical ventilation (p < 0.05) than those in the non-aPRP group. Multiple regression analyses showed that aPRP (odds ratio: 1.729, 95% confidence interval: 1.225–2.440; *p* < 0.001) was one of the independent risk factors for post-AKI.

**Conclusions:**

The use of aPRP significantly reduced intraoperative blood transfusions and decreased postoperative mortality-adjusted mechanical ventilation. However, aPRP use was independently associated with an increased hazard of post-AKI after adjusting for confounding factors.

## Introduction

Stanford type A acute aortic dissection (AAD) is characterized by the rapid development of an intimal flap separating the false and true lumen, blood flow through the nonendothelialized false lumen, and turbulence, which triggers activation of the platelet and coagulation/fibrinolytic system [1, 2]. Moreover, surgery and cardiopulmonary bypass (CPB)-induced coagulation factor consumption and excessive fibrinolysis and platelet activation promote perioperative blood loss and blood product transfusion [3, 4]. Thus, various blood conservation approaches have been applied to improve blood conservation [5–7]. The use of aPRP in cardiac surgery is beneficial for those at high risk of bleeding and reduces blood transfusions during AAD surgery in those undergoing CPB and hypothermic circulatory arrest [8–10]. However, volume replacement and unstable hemodynamics are always along with aPRP harvest process, which is potential risk factors of postoperative acute kidney injury [11]. The purpose of this study was to analyze the effect of aPRP on post-AKI in patients undergoing AAD surgery.

## Materials and methods

### Patient population

**Fig. 1:**
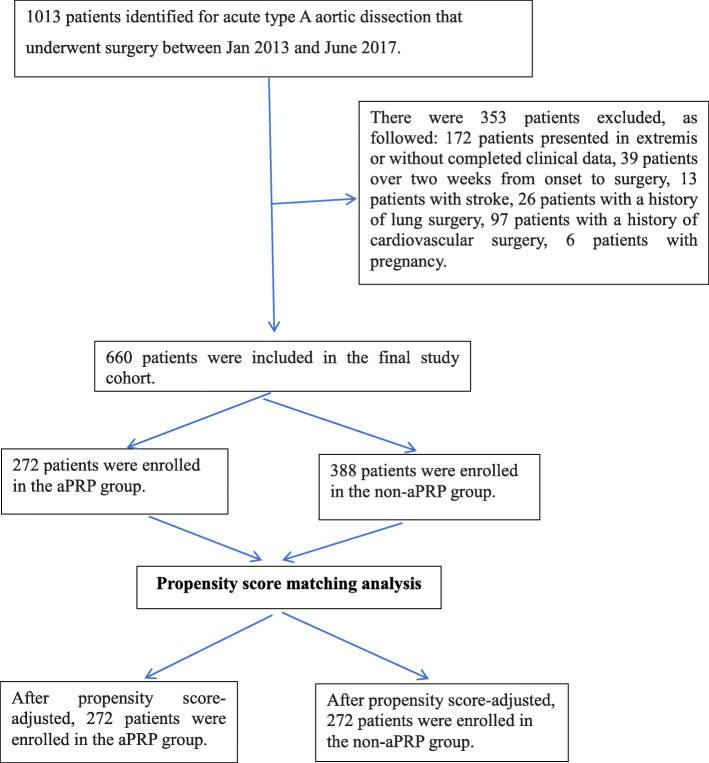
Flow diagram

This study was approved by the Ethics Committee of the Beijing Anzhen Hospital Clinical Research (Beijing, China), and consent was waived because of the retrospective data collection. Patients with Stanford type A AAD were eligible if they were between 18 and 75 years of age and were suitable for emergency surgery. A total of 1013 consecutive patients’ records were collected between January 2013 and June 2017. Patients who had severe cardiac tamponade, cardiogenic shock, cardiac arrest, or severe systolic hypotension were excluded from the analysis for both groups. Exclusion criteria are shown as a flow diagram (Fig. 1). Patients were divided into two groups: aPRP group and non-aPRP group.

### Study endpoint

The primary endpoint of this study was the difference in the incidence of post-AKI after Stanford type A AAD surgery between aPRP group and non-aPRP group. The secondary endpoints were risk factors for post-AKI and to assess clinical outcomes. Clinical outcomes included perioperative blood product transfusions, in-hospital mortality, time to mechanical ventilation, intensive care unit length of stay, and gastrointestinal tract bleeding. Post-AKI was defined as AKI within postoperative 48 h based on Kidney Disease Improving Global Outcomes (KDIGO) criteria [12].

### Surgical technique

After routine anesthesia and intubation, general anesthesia was maintained with intravenous sufentanil, propofol, and neuromuscular blockade drugs. Sun’s surgical technique has been previously detailed [13, 14]. In brief, right axillary artery cannulation was used for cardiopulmonary bypass (CPB) and unilateral selective antegrade cerebral perfusion under moderate hypothermic circulatory arrest at 25 °C. The surgical procedure involved the deployment of a frozen elephant trunk, Cronus (MicroPort Medical, Shanghai, China), into the descending aorta, followed by total arch replacement with a 4-branched vascular graft (Maquet Cardiovascular, Wayne, NJ). Aortic valve or root procedures and concomitant surgeries, such as coronary artery bypass grafting (CABG), were performed during the cooling phase. Blood products were transfused to maintain hemoglobin level > 7 g/L at weaning from CPB, and to correct coagulopathy by thromboelastogram and clinical parameters.

### aPRP harvest technique

aPRP was collected from a large-bore central venous access by gravity drainage and an autologous transfusion system (Sorin-Xtra, Milan, Italy) before the administration of heparin and reinfused after the reversal of heparin. Approximately 15 mL/kg of whole blood was collected. A balanced salt solution (Ringer’s lactate) were used to maintain intravascular volume and hemodynamic stability during the aPRP harvest, as well as continuous IV infusion noradrenaline or dopamine. No cases of hemodynamic instability were noted during aPRP collection.

### Statistical analysis

All data analyses were performed with SPSS 24.0 (IBM, Armonk, NY, USA). Quantitative variables are presented as the mean ± standard deviation or median (interquartile range), and categorical variables are presented as frequencies or percentages. Multiple regression analyses were used to determine the independent factors of post-AKI. Propensity-score matching was performed by logistic regression for variable adjustment, and the variables of age, body mass index (BMI), preoperative SCr, and duration of surgery were used as covariates. Then patients were matched 1:1 based on their propensity scores, with a fixed caliper width of 0.1. All statistical tests were two-sided, and *p* < 0.05 was considered statistically significant.

## Results

### Baseline characteristics

According to the inclusion and exclusion criteria, a total of 660 patients undergoing Stanford type A AAD surgery were ultimately included in this study. The flow diagram is presented in Fig. 1. In the aPRP group, 272 patients (41.2%) received aPRP, whereas 388 patients were in the non-aPRP group. The mean amount of aPRP collected was 594 ± 331 mL, and the mean amount of red blood cells was 428 ± 243 ml. The aPRP and non-aPRP groups demonstrated the differences of age, BMI, history of smoking, preoperative mean blood pressure, hemoglobin, PLC and preoperative SCr (*p* < 0.005) (Table 1).

**Table 1:** Perioperative variables in the non-aPRP group and aPRP group

Clinical variables	Non-aPRP Group(***n*** = 388)	aPRP Group(***n*** = 272)	*P* Value
Age (year)	49.29 ± 10.60	47.52 ± 9.77	0.030
Males, n (%)	305 (78.4)	230 (84.6)	0.067
Height (cm)	172.05 ± 7.51	171.49 ± 8.02	0.359
Weight (kg)	77.02 ± 13.16	78.8 ± 13.20	0.073
BMI (kg/M^2^)	25.97 ± 3.75	26.75 ± 3.70	0.009
History of smoking, n (%)	137 (35.2)	120 (44.1)	0.023
History of hypertension, n (%)	294 (75.8)	199 (73.2)	0.448
History of DM, n (%)	18 (4.6)	13 (4.8)	0.933
Time from onset of symptoms to surgery (d)	2 (1,4)	2 (1, 4)	0.768
**preoperative**			
LVEF (%)	60.22 ± 6.52	60.58 ± 6.302	0.490
LVEDd (mm)	50.76 ± 8.71	49.888 ± 7.8517	0.195
*EuroSCORE*	5 (4.5,5)	5 (5,5)	0.408
HR (beats/min)	80 ± 15	80 ± 15	0.334
MBP (mm Hg)	89 ± 18	85 ± 22	0.014
Hb(g/L)	12.68 ± 2.63	13.91 ± 4.14	0.001
WBC (10^9^/L)	10.91 ± 4.11	11.38 ± 3.72	0.135
PLC(10^9^ /L)	151 ± 76	171 ± 64	0.001
LAC (mmol/L)	1.52 ± 0.95	1.56 ± 0.87	0.549
SCr (umol/L)	102.9 ± 53.1	82.6 ± 37.6	< 0.001
PT (second)	12.74 ± 2.29	12.81 ± 2.06	0.727
PTA(100%)	87.03 ± 14.44	84.67 ± 14.67	0.128
APTT (second)	32.83 ± 11.51	31.70 ± 6.76	0.211
FIB(g/L)	3.30 ± 1.49	3.21 ± 1.56	0.571
FDP (mg/L)	18.65 (9.01,31.45)	19.2 (10.2,35.65)	0.329
D-D (ug/L)	1581 (850, 2976)	1836 (808, 2719)	0.660
INR	1.09 (1.03,1.18)	1.11 (1.04,1.19)	0.192
**Intraoperative**			
With CABG	13 (5.2)	16 (6.5)	0.537
Duration of surgery (min)	465 ± 99	492 ± 105	0.001
Duration of CPB (min)	271.49 ± 172.64	281.27 ± 152.09	0.453
Duration of Aortic cross-clamping (min)	92.97 ± 56.01	86.95 ± 54.77	0.171
Lowest rectal temperature (°C)	25.21 ± 2.13	25.21 ± 1.79	0.966
Lowest nasopharyngeal temperature (°C)	22.46 ± 6.02	22.91 ± 6.60	0.363
Intravenous crystalloid (mL)	2153 ± 887	2354 ± 1133	0.015
Allogeneic Red blood cells (units)	0 (0, 4)	0 (0, 3)	0.012
Allogeneic blood Plasma (mL)	200 (0, 600)	0 (0,400)	0.001
^a^Blood product transfused (ml)	520 (0,1040)	375 (0,975)	0.014
Urine volume during surgery (mL)	1591 ± 1073	1643 ± 863	0.503
**Postoperative**			
LVEF (%)	59.68 ± 7.04	59.59 ± 6.57	0.870
HR (beats/min)	93 ± 17	94 ± 16	0.384
MBP (mmHg)	88 ± 19	86 ± 20	0.20
Hb(g/L)	10.23 ± 1.86	10.42 ± 1.57	0.545
WBC (10^9^/L)	11.48 ± 4.86	12.15 ± 4.57	0.20
PLC(10^9^ /L)	106 ± 51	97 ± 62	0.14
LAC (mmol/L)	2.79 ± 3.12	3.97 ± 3.93	<0.001
PT (second)	13.77 ± 4.45	13.18 ± 2.39	0.080
PTA(100%)	82.81 ± 15.72	81.56 ± 14.86	0.442
APTT (second)	32.63 ± 7.13	33.10 ± 12.04	0.628
FIB(g/L)	3.34 ± 1.43	3.39 ± 1.97	0.731
FDP (mg/L)	19 (9.17,32.4)	17.56 (9.52,35.67)	0.929
D-D (ug/L)	1963 (855,3046)	1619 (815,2841)	0.510
INR	1.12 (1.04,1.24)	1.14 (1.06,1.24)	0.323
SCr (umol/L)	118.58 ± 72.59	105.89 ± 51.50	0.011
**Postoperative 24 h**			
SCr (umol/L)	133.1 ± 91.8	115.2 ± 66.6	0.005
Seroma volume of drainage (mL)	1472 ± 667	1539 ± 820	0.263
**Postoperative 48 h**			
SCr (umol/L)	136.1 ± 106.3	122.3 ± 98.2	0.102
**Postoperative 72 h**			
SCr (umol/L)	113.4 ± 93.7	110.1 ± 77.5	0.737

Data are given as numbers, percentage, mean ± standard deviation or median (interquartile range, IQR). *BMI* Body mass index, *DM* Diabetes mellitus, *LVEF* Left ventricular ejection fraction, *LVEDD* Left ventricular end diastolic diameter, *EuroSCORE* European system for cardiac operative risk evaluation, *MBP* Mean blood pressure, *PLC* Platelet count, *Hb* Hemoglobin, *WBC* White blood cells, *LAC* Lactic acid, *FIB* Fibrinogen, *FDP* Fibrinogen degradation product, *APTT* Active part thrombin time, *PT* Prothrombin time, *APTT* Activated partial thromboplastin time, *PTA* Prothrombin activityprothrombin time activity, *INR* International normalized ratio, *D-D* D-Dimer, *SCr* Serum creatine, *CABG* Coronary artery bypass grafting, *CPB* Cardiopulmonary bypass. ^a^Number of blood products transfused perioperatively was defined as total units of red blood cells (1 unit = 160 ml), fresh-frozen plasma and apheresis platelet units (1 unit = 260 ml) administered

### Intraoperative

Duration of surgery was more longer in the aPRP group than in the non-aPRP group. The aPRP group required fewer intraoperative transfusions (*p* < 0.001) and more intravenous crystalloid (*p* < 0.05) than the non-aPRP group. Adjustment for propensity score did not materially alter the clinical effects and statistical differences for these intraoperative blood products and intravenous solution.

### Postoperative outcomes

Postoperative outcomes are shown in Table 2. The primary association of aPRP with unadjusted postoperative complications involved less mortality-adjusted mechanical ventilation (*p* = 0.029) and in-hospital mortality (*p* = 0.001). Upon propensity score adjustment, in-hospital mortality was similar in both groups (*p* = 0.904). The occurrence of post-AKI was greater in the aPRP group, both without and with propensity adjustment (14.1% (*p* = 0.002) and 11.1% (*p* = 0.010), respectively). Multiple regression analyses of these variables showed that aPRP (odds ratio [OR]: 1.729, 95% confidence interval [CI]: 1.225–2.440; *p* < 0.001), postoperative Scr (OR: 1.010, 95% CI: 1.006–1.014; *p* < 0.001) and postoperative LAC (OR: 1.104, 95% CI: 1.047–1.164; *p* < 0.001) were significantly associated with post-AKI with adjustment for factors (Table 3). The AUROC (receiver operating characteristic) of this multivariable binary logistic regression analysis was 0.790 (95% CI, 0.754–0.825).

**Table 2:** Postoperative complications compared between non-aPRP and aPRP groups

Variables	Unmatched			Propensity score-matched		
	Non-aPRP Group (***n*** = 388)	aPRP Group (***n*** = 272)	***P*** Value	Non-aPRP Group (***n*** = 272)	aPRP Group (***n*** = 272)	***P*** Value
Mortality-adjusted Mechanical ventilation (h)^a^	40 (17,95)	28 (16,67)	0.029	40 (17,92)	28 (16,67)	0.043
ICU length of stay(h)^a^	45 (23,90)	43.5 (21.5,95.5)	0.439	40 (22,82)	43.5 (21.5,95.5)	0.368
Post-AKI, n (%)	152 (39.2)	145 (53.3)	0.002	115 (42.2)	145 (53.3)	0.010
CRRT, n (%)	11 (2.8)	25 (9.2)	0.013	10 (3.8)	25 (9.2)	0.015
GI tract bleeding, n (%)	39 (10.1)	34 (12.5)	0.324	34 (12.8)	34 (12.5)	0.922
In-hospital mortality, n (%)	37 (9.5)	16 (5.9)	0.001	15 (5.6)	16 (5.9)	0.904

^a^ In-hospital Survivors; *ICU* Intensive care unit, *CRRT* Continuous renal replacement therapy, *post-AKI* Acute kidney injury within postoperative 48 h, *GI* Gastrointestinal; Other abbreviations are presented in Table 1

**Table 3:** Risk factors for postoperative differences stage acute kidney injury after repair of acute Type A aortic dissection in the multivariate-adjusted model ^a^ (*n* = 660)

Variables	Post-AKI		
	OR	95% (CI)	***P*** Value
**aPRP**	**1.729**	**1.225–2.440**	**0.002**
**postoperative serum creatine (umol/L)**	**1.010**	**1.006–1.014**	**< 0.001**
**Postoperative LAC (mmol/L)**	**1.104**	**1.047–1.164**	**< 0.001**

*OR* odds ratio, *CI* confidence interval

^a^ adjusted for male, age, BMI, Intravenous crystalloid, Blood product transfused, Duration of surgery, and History of smoking

**Fig. 2:**
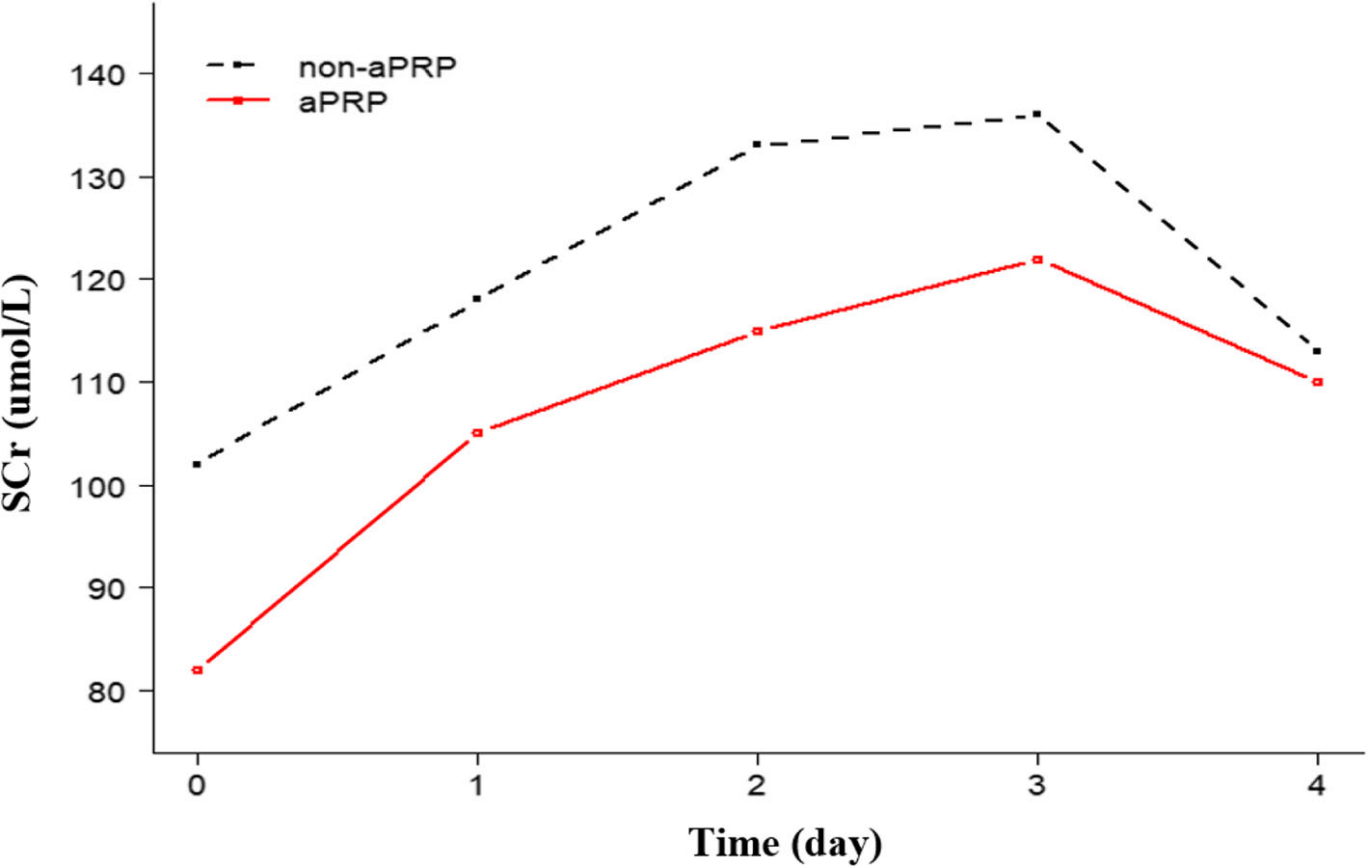
Perioperative SCr changes from preoperative to postoperative 3-day time points in both groups. 0, preoperative; 1, postoperative; 2, postoperative 1 day; 3, postoperative 2 days; 4, postoperative 3 days

Figure 2 depicts perioperative SCr continuously increasing from preoperative to postoperative 3-day time points in the two groups (*p* < 0.001). We repeated measurements of perioperative SCr at each follow-up visit to characterize the changes in post-AKI over time in both groups. SCr levels increased and peaked at 2 days after surgery and then decreased until 3 days after surgery in all patients. There was no significant differences in treatment-time effects for perioperative SCr levels between both groups. However, in the first period (from preoperative to postoperative), there was a mean maximal increase in SCr of 15.6 and 28.1% in the non-aPRP and aPRP groups, respectively; in the last period (postoperative 2–3 days), there was a mean maximal decrease in SCr of 16.9 and 9.8% in the non-aPRP and aPRP groups, respectively.

## Discussion

Theoretically, aPRP is less exposure of coagulation factors and platelets to the extra-circulation system to achieve blood conservation from the deleterious effects of CPB. The centrifugation device and autologous transfusion system can be used to harvest and separate whole blood into red cell concentrate and aPRP fraction. The collected red cell is reinfused depended on hemoglobin concentration, and aPRP fraction is reinfusion after separation from CPB and reversal of anticoagulation.

Similar to prior reports [8, 10, 15], we found the aPRP technique markedly reduced intraoperative allogeneic blood transfusion and shortened the duration of mechanical ventilation. For whole blood exposed to the CPB circuit, the patient’s platelets were activated, and coagulation factors were consumed during CPB. The use of aPRP can maintain normal platelet function, preserve plasma volume, and ultimately reduce transfusion. In previous studies, Zhou [8] and Han [15] presumed that the use of aPRP could ameliorate postoperative lung injury and shorten mechanical ventilation, possibly related to fewer allogeneic transfusions.

AKI is frequent as a severe complication following an operation for Type A AAD. According to KDIGO criteria, the reported incidence of post-AKI after thoracic aortic surgery is 53% [16] and 77.6% [17] in China. In this study, we found that there was a significantly lower incidence of post-AKI (45%) compared with the results reported previously. In the present study, we defined post-AKI as AKI occurred within 48 h after surgery, which reduced the incidence of post-AKI and avoided other postoperative complications to confounding the effect of aRPR on renal function.

In this study, the use of aPRP was independently associated with an increased hazard of post-AKI. The main risk with aPRP harvest was hemodynamic fluctuation and hemodilution induced by acute hypovolemia and alternate fluid therapy. In the aPRP group, rapid fluid replacement therapy were required to maintain hemodynamic stability during aPRP collection. Total fluid volume for transfusion was higher in the aPRP group than in the non-aPRP group, which not related to post-AKI by Logistic analysis.

In this study, post-AKI development after type A acute aortic dissection repair was associated mainly with postoperative variables, such as postoperative serum creatinine and lactate. Although novel biomarkers, such as neutrophil gelatinase-associated lipocalin and cystatin C, have been identified as independent predictors of AKI and are superior to conventional biomarkers, sCr continues to be a more valuable and accepted tool for AKI diagnosis [18]. During surgery and CPB, the kidneys may suffer from an imbalance between oxygen supply and oxygen needs that is associated with lactate production [19]. Higher serum lactate values, which are a surrogate marker of tissue hypoperfusion and imbalance of renal oxygen metabolism, were associated with the occurrence of postoperative AKI. In addition, lower hemoglobin induced by hemodilution during aPRP collection decreased oxygen delivery and was also associated with postoperative AKI [20].

The precise mechanism of aPRP on post-AKI has not been clarified. Thus, careful monitoring and management of hemodynamic and maintaining fluid balance by experienced anesthesiologists during aPRP harvest play a vital role in improving postoperative kidney function. In addition, there still are some questions to be addressed for the application of aPRP in future studies.

### Limitations

This retrospective study has several limitations. The most important limitation was the difference in preoperative variables between groups and the possibility of selection bias due to the nonrandomized design. The preoperative characteristics of patients in the aPRP group were younger age, higher BMI values, lower levels of preoperative SCr, and longer duration of surgery patients than those in the non-PRP group. The characteristics between the two groups could be adjusted to partially correct for these differences by propensity-adjusted matching analysis. Additionally, preoperative renal malperfusion is an independent predictor for postoperative AKI [21]. Preoperative renal malperfusion also induced an increase in preoperative SCr values, which possibly led to bias and adverse effects on the results. In this study, preoperative SCr values were higher in 160 (160/660, 24.2%) patients than after surgery, and only 25 (25/160, 15.6%) of these patients had post-AKI. Thus, instead of focusing on preoperative SCr, postoperative SCr was selected in our model of risk factors associated with post-AKI. Ultimately, most patients with severe ischemia or malperfusion were not enrolled in this study. The results were appropriate for patients classified as Penn class Aa but are not a guideline to those who are Penn class Ab and Ac [22].

## Conclusion

The use of aPRP significantly reduced intraoperative blood transfusions, as well as decreased postoperative mortality-adjusted mechanical ventilation in patients undergoing open repair of acute Type AAD. However, aPRP was independently associated with an increased hazard of post-AKI after adjusting for confounding factors.

## Data Availability

The datasets used and/or analysed during the current study are available from the corresponding author on reasonable request.

## Abbreviations

AAD: Stanford type A acute aortic dissection
aPRP: Autologous platelet-rich plasmapheresis
post-AKI: Postoperative acute kidney injury
CPB: Cardiopulmonary bypass
KDIGO: AKI based on Kidney Disease Improving Global Outcomes
Scr: Serum creatinine
BMI: Body mass index
DM: Diabetes mellitus
LVEF: Left ventricular ejection fraction
LVEDD: Left ventricular end diastolic diameter
EuroSCORE: European system for cardiac operative risk evaluation
MBP: Mean blood pressure
PLC: Platelet count
Hb: Hemoglobin
WBC: White blood cells
Glu: Glucose
LAC: Lactic acid
FIB: Fibrinogen
FDP: Ibrinogen degradation product
APTT: Active part thrombin time
PT: Prothrombin time
APTT: Activated partial thromboplastin time
PTA: Prothrombin activityprothrombin time
ICU: Intensive care unit
CRRT: Continuous renal replacement therapy
AKI: Acute kidney injury
GI: Gastrointestinal
OR: Odds ratio
CI: Confidence interval
